# Impact of scheduling multiple outdoor free-play periods in childcare on child moderate-to-vigorous physical activity: a cluster randomised trial

**DOI:** 10.1186/s12966-018-0665-5

**Published:** 2018-04-04

**Authors:** Lubna Abdul Razak, Sze Lin Yoong, John Wiggers, Philip J. Morgan, Jannah Jones, Meghan Finch, Rachel Sutherland, Christophe Lecathelnais, Karen Gillham, Tara Clinton-McHarg, Luke Wolfenden

**Affiliations:** 1 Hunter New England Population Health, Wallsend, NSW 2287 Australia; 2 School of Medicine and Public Health, University of Newcastle, Callaghan, NSW 2308 Australia; 3 School of Education, Priority Research Centre in Physical Activity and Nutrition, University of Newcastle, Newcastle, NSW Australia; 4 School of Psychology, University of Newcastle, Newcastle, NSW 2308 Australia; 5 Hunter Medical Research Institute, Newcastle, NSW 2300 Australia; 6 Priority Research Centre for Health Behaviour, The University of Newcastle, Callaghan, NSW 2308 Australia

**Keywords:** Child day care services, Preschool, Childcare, Young children, Physical activity, Outdoor, Scheduling, Intervention, Randomised controlled trial

## Abstract

**Background:**

Increasing the frequency of periods of outdoor free-play in childcare may represent an opportunity to increase child physical activity. This study aimed to assess the efficacy of scheduling multiple periods of outdoor free-play in increasing the time children spend in moderate-to-vigorous physical activity (MVPA) while attending childcare.

**Methods:**

The study employed a cluster randomised controlled trial design involving children aged 3 to 6 years, attending ten childcare services in the Hunter New England region of New South Wales, Australia. Five services were randomised to receive the intervention and five to a control condition. The intervention involved services scheduling three separate periods of outdoor free-play from 9 am to 3 pm per day, each at least 15 min in duration, with the total equivalent to their usual daily duration of outdoor play period. Control services implemented the usual single continuous period of outdoor free-play over this time. The primary outcome, children’s moderate-to-vigorous physical activity (MVPA) while in care per day, was measured over 5 days via accelerometers at baseline and at 3 months post baseline. Secondary outcomes included percentage of time spent in MVPA while in care per day, total physical activity while in care per day and documented child injury, a hypothesised potential unintended adverse event. Childcare services and data collectors were not blind to the experimental group allocation.

**Results:**

Parents of 439 (71.6%) children attending participating childcare services consented for their child to participate in the trial. Of these, 316 (72.0%) children provided valid accelerometer data at both time points. Relative to children in control services, mean daily minutes of MVPA in care was significantly greater at follow-up among children attending intervention services (adjusted difference between groups 5.21 min, 95% CI 0.59–9.83 *p* = 0.03). Percentage of time spent in MVPA in care per day was also greater at follow-up among children in intervention services relative to control services (adjusted difference between groups 1.57, 95% CI 0.64–2.49 *p* < 0.001). Total physical activity while in care per day, assessed via counts per minute approached but did not reach significance (adjusted difference between groups 14.25, 95% CI 2.26–30.76 *p* = 0.09). There were no differences between groups in child injury nor subgroup interactions for the primary trial outcome by child age, sex, or baseline MVPA levels.

**Conclusion:**

Scheduling multiple periods of outdoor free-play significantly increased the time children spent in MVPA while in attendance at childcare. This simple ecological intervention could be considered for broader dissemination as a strategy to increase child physical activity at a population level.

**Trial registration:**

This trial was prospectively registered with the Australian New Zealand Clinical Trials Registry (ANZCTR) (ACTRN1261000347460). Prospectively registered 17th March 2016.

**Electronic supplementary material:**

The online version of this article (10.1186/s12966-018-0665-5) contains supplementary material, which is available to authorized users.

## Background

Sufficient physical activity in early childhood (under 6 years) can accrue immediate metabolic benefits in blood pressure and lipid profile and reduce the risk of unhealthy weight gain [1]. Adequate physical activity is also associated with social, emotional, cognitive [2], and motor skill development [3]. Furthermore, physical activity in early childhood tracks into adulthood [4]. Despite this, just 41.6 to 50.2% of preschool-aged children in the US [5] and 10% in Australia [6] currently meet recommended levels of at least 15 min physical activity per hour while in care [7], measured objectively.

Childcare services are a key setting in which to intervene to improve physical activity levels given that they provide access to a large number of children [8] for prolonged periods. In Australia, children in long day care spend 20 h each week on average, with 43% attending three to 5 weekdays and 57% attending just one to 2 weekdays [9]. For preschools, children attend an average of only 13 h a week with 54% attending 1–2 weekdays in care. Long day care services provide centre-based care for eight or more hours per day for 5 days a week and typically enrol children from 6 weeks to under 6 years [10]. Preschools provide centre-based care for 6 to 8 h per day and enrol children between 3 and 6 years. Childcare services also have infrastructure that can be utilised to create environments supportive of physically active play via outdoor space and equipment which cater to children’s varying activity interests [11].

A recent meta-analysis of randomised trials of childcare-based physical activity interventions reported that their effectiveness was equivocal [12]. The review identified poor implementation of multi-component and complex interventions requiring staff training and resources as a potential contributing factor [13–15]. One potential opportunity to improve the impact of physical activity interventions in the childcare setting may be to design interventions that are more likely to be implemented. Previous research has established that preschool children’s activity is characterized by short intense bouts of activity between 3 and 15 min occurring at the start of periods of outdoor free-play, followed by extended recovery periods of sedentary behaviour or light activity [16–19]. Increasing the frequency of outdoor free-play opportunities may, therefore, capitalise on the natural tendency for children to be active at the start of outdoor free-play periods [16, 19]. Furthermore, incorporating such changes into childcare service scheduling and programming may not require additional skills, training or expensive resources to implement, frequently reported barriers to the delivery of other physical activity interventions in this setting [20].

A recently published study assessed the effect of scheduling more frequent periods of free-play, as part of a multi-component intervention, on children’s physical activity levels and sedentary time in care [21]. Specifically, intervention services scheduled four 30 min periods of outdoor free-play, with trained educators in physical activity promotion, during which additional portable equipment such as balls, hula hoops, hopscotch mats, obstacle courses, stepping domes, ribbon wands and hop along bouncers was also made available. The 8 week intervention was found to be effective while the more frequent outdoor free-play periods were implemented as scheduled, but not at 12 months follow-up; when services were observed to have ceased their implementation. Furthermore, being multi-component, this trial was unable to delineate which components of the trial had been effective in improving child activity.

Given the promising effects observed for outdoor free-play period scheduling, in combination with trained staff and equipment provision, the current study sought to extend the evidence base and isolate the effectiveness of repeated periods of outdoor free-play opportunities on child physical activity. Specifically, the aim of the study was to assess the efficacy of scheduling three periods of outdoor free-play each day in childcare services in increasing the time children spend in MVPA when attending childcare, compared to a period of continuous play of equal duration.

## Methods

The trial is reported in accordance with the CONSORT statement and its extension on cluster randomised trials [22]. A detailed protocol for this trial has been previously published [23].

### Design and setting

The study employed a between group, cluster randomised controlled trial design (see Table 1). Ten centre-based childcare services, with only one scheduled period of outdoor free-play during their core operating hours of 9 am to 3 pm (of at least 45 min duration) were randomised to an intervention or control group (1:1 ratio). Services were selected from the Hunter New England region of New South Wales, Australia. The intervention was 3 months in duration. Data on child physical activity during care were assessed on a cohort of children, via accelerometer over a 5-day period at baseline and immediately post intervention at approximately 3 months post baseline.

**Table 1:** Illustration of the flow of trial

3 weeks	5–7 days	3 months	5–7 days
Recruitment -Services (verbal) -Parent (informed consent) -Child (verbal)	Baseline data collection NS Interviews In-care & out of care accelerometry EPAO Parent CATI	After randomisation, intervention services – 3 outdoor free-play periods; control services – maintain 1 continuous free-play period	Follow-up data collection In-care & out of care accelerometry EPAO

### Participants and recruitment

#### Childcare services

To be eligible to participate in the trial, services were required to have a daily enrolment of at least 25 children aged 3 to 6 years. Services also needed to have an existing schedule of outdoor free-play time for children consisting of a single period of at least 45 min during the core hours of service from 9 am to 3 pm. Services that reported already having more than one outdoor free-play period were ineligible to participate in the trial. Services catering solely for occasional care or children with special needs (e.g. requiring specialist support, which may affect physical activity scheduling) were excluded from the trial as were services currently participating in other interventions trials within the study region (nutrition and educator trials).

Recruitment was conducted from April to June 2016. A member of the research team, who was not involved in the delivery of the trial or data collection, made telephone contact with childcare services to assess eligibility, and invited eligible services to participate in the study. Once verbal consent was obtained, services were invited to take part in a short telephone interview. Study information forms and consent forms were sent to the services to distribute to parents of eligible children enrolled at consenting childcare services (14 out of a potential 219 services) across the study region.

The trial originally sought to utilise probability-sampling methods to recruit childcare services; however a change in recommended practice for the setting (that services provide ongoing rather than structured opportunities for outdoor free-play across the day) meant that a large proportion of services (58%) were ineligible for the current study [24]. As such, a convenience sample of 14 eligible services were identified and consented to participate. A further four services were deemed ineligible at baseline due to not having one period of outdoor free-play in core hours of 9 am to 3 pm.

#### Parents and children

To be eligible to participate in the data collection component of the study, children were required to be aged 3 to 6 years and, to have attended participating services between 9 am to 3 pm on 1 or more days during the week of data collection. During the week prior to the agreed week of baseline data collection, a research assistant was also deployed during periods of drop-off or pick-up of children to distribute information and consent forms to parents at each service. Parents were invited to provide consent for their child to participate in measurement i) at childcare and ii) at home (using accelerometers). Parents could consent to children wearing accelerometers in care but not at home. All parents of participating children were also invited to participate in a computer -assisted telephone interview (CATI).

### Randomisation, allocation and blinding

A statistician with no other involvement in recruitment or data collection allocated services to either the intervention or the control condition in a 1:1 ratio using a computerised random number generator, following baseline data collection. Randomisation of childcare services were stratified by the socioeconomic status of the areas where the services were located (using their postcode), and the service type (long day care service or preschool) based on previous finding of an association between these factors and the physical activity policies and practices of services [25]. Services were informed of their experimental group allocation after baseline data collection by a member of the research team. Data analysts were blinded to the group allocation of intervention and control services.

#### Intervention

The intervention sought to create a childcare environment supportive of child physical activity by scheduling multiple opportunities for outdoor free-play in a way that is consistent with a child’s natural physical activity patterns [26, 27]. Specifically, within a 6 h day (9 am to 3 pm), the intervention involved dividing the single usual period of outdoor free-play from children into three periods of at least 15 min duration per period. For example, an intervention service, which usually scheduled one 60 min continuous free-play period, was rescheduled to two shorter periods in the morning of 15 min each, and one period in the afternoon of 30 min. Services were encouraged to keep the total duration of outdoor free-play across the day consistent with that assessed at baseline.

Immediately following baseline data collection services allocated to receive the intervention were contacted by a member of the research team and an early childhood education specialist to support the implementation of the intervention. All services were asked to accept two visits and two telephone calls to their service to assess if implementation was taking place and provide the opportunity to give feedback to those services experiencing any difficulties. Services were also offered written materials covering national guidelines on physical activity, “Get up & Grow” materials, Sun Smart Shade manual, benefits of outdoor play resources from the Raising Children network, relevant National Quality Standard professional learning newsletters. A standardised recording template were used to record the delivery of a site visit, telephone contacts and resources (if any) supplied to the service to support implementation.

#### Control

Services allocated to the control group were asked to continue to schedule their usual single period of outdoor free-play in the core hours of 9 am to 3 pm. Control services agreed not to make any changes to the total duration of this single continuous period throughout the duration of the study. No other support was offered to control services during the study period.

### Data collection procedures and measures

Baseline data collection was conducted between May and July 2016 (autumn/fall–winter season) and follow-up data collected 3 months later (August–November 2016; winter–spring season). (Table 1).

#### Parent and child characteristics

At baseline, parents, provided brief demographic information on the child’s consent form, including the child’s date of birth and sex. Other data collected included the number of days the child attended the childcare service each week and their residential postcode to assess the socioeconomic status of their usual place of residence.

In addition, a computer-assisted telephone interview (CATI) was conducted with consenting parents to collect: child and parent demographic information (parent age, parent sex, Aboriginal and/or Torres Strait Islander status, household income and parent education); usual levels of parent physical activity; and child and parent weight and height, using items from the New South Wales Population Health Survey [28].

#### Services characteristics

During recruitment, a baseline telephone interview was conducted with supervisors of participating childcare services that assessed the following: postcode (to assess the socioeconomic status of the area) [29], number of years’ the service has been in operation and the total number of 3 to 6 year-old children enrolled.

#### Service outdoor free-play schedule and physical activity environment

Observations at childcare services were conducted by pairs of trained data collectors to record the duration (via stopwatch), timing and frequency of outdoor free-play to ensure that services were implementing outdoor free-play periods according to the study protocol.

The two data collectors also collected information regarding the broader childcare service physical activity environment and educator physical activity practices using a comprehensive assessment tool (Environment and Policy assessment and observation instrument, EPAO) [30]. This information was collected daily over the 5 day data collection period at baseline and at follow-up. EPAO assessment conducted over 5 days have been shown to provide more reliable estimates of usual childcare environments than those conducted over a single day [31]. The following types of physical activity observation elements were assessed as part of the EPAO: active play opportunities, sedentary opportunities, sedentary environment, portable play environment, fixed play environment, staff behaviours (e.g. prompts and positive statements), physical activity training, education, and existence of a written physical activity policy. These items are used to calculate a sub-score and an overall score. Other data collected included the number of children in attendance, number of room staff working on the days of data collection, outdoor play area size (m ^2^), and minimum and maximum daily temperatures [32] and UV index [33].

#### Child physical activity

Accelerometers (Actigraph GT3X+) were used to collect information on child physical activity. The accelerometers were worn by children from the time they first arrived at the childcare service until 3 pm on each day of attendance. Accelerometer data were collected on every day of 1 week (5 days in total) of the data collection period at baseline and follow-up. Two data collectors (not blinded to experimental group allocation) attended the services during the data collection period to fit and collect the accelerometers using a standard protocol. Accelerometers were placed above the left iliac crest at the hip of the child using a clip or band. Children with at least 50% of wear time during childcare hours on 1 day/week were considered to have valid wear time. All participating children wore an ‘in care’ accelerometer each day (up to 5 days) that they attended care. Data from children consenting to also wear an additional accelerometer ‘out of care’, was used for descriptive purposes to assess any potential compensatory effect in children’s physical activity during out of care periods on days children attend care.. These children had their ‘in care’ device removed at 3 pm on each day of attendance or earlier if they left the service for the day, but kept on wearing the ‘out of care’ device. Data collectors also recorded if children removed accelerometers during naps or other times when the belt was removed. On the first day of wearing for home, parents were reminded of their agreement to keep a daily log of their child’s activities, when they did not wear the device, and periods of sleep.

#### Primary outcome

The primary trial outcome was the mean daily minutes that children spent in MVPA from the time of arrival at the service until 3 pm, over the course of 1 week (5 days) and for every day of care attendance (ranging from 1 to 5 days). Minutes of MVPA were assessed using recommended cut points [34]. The Actigraph accelerometer has established utility, validity, and reliability and is the current gold standard for assessment of activity in children aged 3 to 6 years [35].

#### Secondary outcomes

Secondary trial outcomes included total child activity (counts per minutes in 5 s epochs) while in care per day [36], and percent of time children spent in MVPA adjusted for wear time per day, as assessed by accelerometer. Counts per minute were calculated from the total activity counts recorded divided by the total time the accelerometer was worn (1440 × number of valid days).

To identify any potential adverse effect of the intervention, the number of injuries requiring documentation during the past 3 months was assessed during interviews with childcare services’ supervisors, at baseline and at follow-up using items taken from a previous childcare physical activity study conducted by the research team [14].

#### Sample size and power calculations

The study aimed to approach approximately 500 children from 14 childcare services across the study region. We assumed a standard deviation of MVPA of 2.7 min/h [37] and an intra-class correlation coefficient of 0.1 [38], that a sample of 14 children per cluster (assuming a conservative participation rate of approximately 50% and a 20% loss to follow-up) would provide the study with 80% power to detect a change of 9.9 min in daily MVPA. An increase in 10 min of MVPA in children aged 3 to 6 years have been found to have clinically significant beneficial effects on fat mass [39] and peak bone mass [40].

### Statistical analyses

All statistical analyses were performed using SAS (version 9.3) statistical software. All statistical tests were two tailed with an alpha value of 0.05. Summary statistics were used to describe all variables of interest. Accelerometer data were cleaned using the Meterplus software. Twenty minutes of consecutive, 0 min were classified as non-wear and eligible data for in-care periods was based on a least 50% of wear time during the school day. Invalid wear days were removed from the analysed dataset. Generalised Linear Mixed Models (GLMM), to take into account the clustering of individual children within services, were used for primary and secondary physical activity outcomes. An intention to treat framework was used to test a mean difference between groups after 3 months, while adjusting for baseline assessment of outcomes. Each GLMM also controlled for child age, sex and total outdoor free-play duration at follow-up. Analyses entailed multiple imputation for missing data [41] and also performed first using all available (complete case analysis) data without multiple imputation. Pre-specified subgroup analysis for the primary trial outcome was undertaken by child age, sex and baseline activity levels (classified as more or less active based on the median MVPA value of children at baseline). This was undertaken to assess differential changes between groups by introducing a group by subgroup interaction term into the models.

To assess any compensatory changes in physical activity which occurred outside the hours of care as a result of the intervention, average daily minutes of MVPA for out of care periods (for the days the child attended the service) were also analysed. Differences in adverse events over time were assessed using a non-parametric test (Wilcoxon rank-sum test) comparing by group at follow-up for the mean number of reported child injuries per service.

## Results

### Sample

**Fig. 1:**
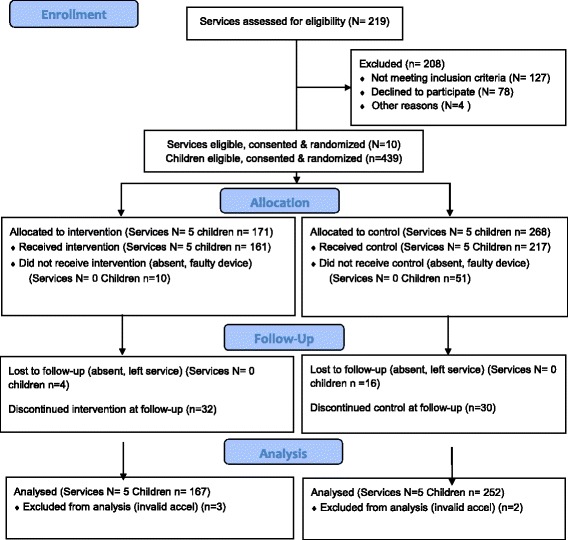
Participant recruitment and retention by group

From the ten participating services, consent was obtained from 439 (71.6% of enrolled children). At baseline, 378 children had valid data (86.1% of consenting children) children (Fig. 1). At follow-up, 357 children (81.3% of consenting children) had their physical activity assessed via accelerometer and found to have adequate wear time. At the child level there was 2.3% loss to follow-up in intervention group services and 6.0% loss to follow-up in control group services. The primary reasons for loss to follow-up were absences, refusal to wear an accelerometer, and faulty accelerometers (Additional file 1).

At baseline, 161 (total wear time in care – 813.7 min (sd. 373.3) and 217 (total wear time in-care – 724.4 min (sd. 334.3) children provided valid data in the intervention and control services respectively. At both time points, adequate wear time for analysis was provided for 167 (96.4% of children wearing accelerometer at baseline) children in the intervention services and 252 (86.8% of children wearing accelerometer at baseline) children in the control services (Fig. 1).

For analyses of out of care physical activity to assess any compensatory effects, valid accelerometer data was available for 33 (70.2% of children wearing an out of care accelerometer) children in the intervention services and 100 children (67.6% of children wearing an out of care accelerometer) in the control services.

Of those who had valid accelerometer data at baseline, 244 out of 282 (64.6%) consenting parents/guardians completed the telephone survey.

#### Parent and child characteristics

The characteristics of participating children were similar at baseline for most characteristics (including age, sex, Aboriginal and/Torres Islander status, body mass index (BMI) (Table 2). The mean days of childcare attendance for children attending in the intervention services did not differ than for children attending control services. There were a higher proportion of families in the control services with a higher household income and with a parent with a university education.

**Table 2:** Child, parent, and Service characteristics by group at baseline

	Intervention	Control
Child characteristics		
No of children^a^	161	217
Age of child; mean (years, sd.)	3.73 (0.59)	3.80 (0.68)
Male, *n* (%)	92 (57.14)	110 (50.69)
Aboriginal and Torres Island Status^b^ (*n*, %)	17 (18.08)	21 (14.00)
BMI^b^ in kg/m ^2^, mean (sd.)	18.07 (5.10)	16.28 (2.12)
Days per week the child usually attends, mean (sd.)	2.75 (0.92)	2.40 (0.88)
Usual residence socio-economic area (*n*, %)		
Upper 50% of New South Wales	99 (61.49)	71 (33.18)
Lower 50% of New South Wales	62 (38.51)	143 (66.82)
Parent characteristics		
Number of parents^c^	95	150
Mother (*n*, %)	81 (85.26)	131 (87.33
Age 30–39 years (*n*, %)	54 (56.84)	80 (53.33)
Country of birth (Australia) n,%	92 (96.84)	138 (92.00)
Consenting parent had university qualifications, *n* (%)	33 (34.74)	79 (52.67)
Parent income > $80 K per year, *n* (%)	55 (58.51)	107 (71.81)
Usual physical activity (PA) (meeting national PA guidelines), *n* (%)	38 (40.43)	62 (41.89)
Service Characteristics		
Number of services	5	5
Service Type; Long Day Care, *n* (%)	4 (80)	2 (40)
Years of operation, mean (sd.)	19.67 (17.22)	16.35 (19.01)
Service geographical location (*n*, %)		
Urban	2 (40.00)	3 (60.00)
Rural	3 (60.00)	2 (40.00)
Service socio-economic area (*n*, %)		
Upper 50% of New South Wales	2 (40.00)	1(20.00)
Lower 50% of New South Wales	3 (60.00)	4 (80.00)
Children aged 3–6 years enrolled – overall, mean (sd.)	54.8 (6.26)	80 (16.09)
No of primary contact staff, mean (sd.)	2.18 (0.46)	2.75 (1.16)
Outdoor play area in m^2^, mean (sd.)	634.95 (226.01)	458.00 (152.15)
Median (min, max)	689.3 (306.44, 927.68)	467.23 (251.61, 698.79)

All measured at baseline

^a^All children who had valid in care accelerometer data at baseline

^b^Denominator is children who had a parent complete the baseline computer-assisted telephone interview

^c^Parents of children who had valid accelerometer data at baseline

#### Services characteristics

Service characteristics by intervention and control group are shown in Table 2. Four out of five intervention services were long day care as were two out of five control services. Intervention services had a larger median outdoor play area compared to control services.

### Intervention fidelity

At baseline, one of the five control services had 4 days out of five valid days of data collected, due to inclement weather. All five intervention services scheduled their outdoor play on each of the 5 days of data collection. At follow-up, intervention services had a total of 20 days of data collection days whereas control services had 21 days.

Among intervention services, two services received two site visits by the research team and three services received a single site visit. Four services received two telephone support calls and one service did not receive any telephone support contact. None of the services were interested in receiving additional implementation support resources. In four of the five services, full implementation of the scheduling intervention occurred prior to follow-up data collection. This was verified from site visits and telephone contacts. One service only implemented the intervention for the week of data collection (at 3 months).

### Outdoor free play duration and physical activity environment

The average total outdoor free play duration in the control childcare services was 160.98 min (sd. 76.19) per day at follow-up. In the intervention services, the average total outdoor free-play duration was 103.13 min (sd. 35.86) per day at follow-up.

Analysis of the EPAO total scores found no significant changes over time in measures of the physical activity environment (adjusted difference 0.66 (95% CI −4.18–2.86, *p* = 0.68). Specifically, the mean total physical activity environment score was 12.30 (sd. 2.38) at baseline and 12.13 (sd. 2.04) at follow-up for intervention services. For the control services, the baseline and follow-up mean total physical activity environment score for the control services were 12.74 (sd. 3.07) and 12.78 (sd. 2.73) respectively (Table 3).

**Table 3:** Changes in EPAO scores and weather from baseline to 3 month follow-up

	Intervention		Control		Intervention–control	
	Baseline Mean *n* = 38	Follow-up Mean *n* = 31	Baseline Mean *n* = 48	Follow-up Mean *n* = 41	Adjusted difference between group (95% CI)	*p*-value
*Physical Activity Environment Total Score*	12.30 (2.38)	12.13 (2.04)	12.74 (3.07)	12.78 (2.73)	0.66 (−4.18–2.86)	0.68
*Physical activity environment subscales*						
*Active Opportunities*	12.24 (4.02)	14.03 (3.96)	12.92 (2.93)	12.74 (4.53)	1.11 (−4.73–6.95)	0.67
*Sedentary Opportunities*	21.17 (3.87)	19.25 (4.53)	18.22 (5.06)	18.86 (4.81)	0.59 (−4.14–5.32)	0.78
*Sedentary Environment*	13.33 (0.00)	17.33 (3.65)	13.33 (9.43)	9.33 (7.60)	8.00 (−0.70–26.70)	0.07
*Portable Play Environment*	11.43 (2.86)	9.71 (3.83)	12.00 (4.69)	11.43 (4.52)	−1.71 (−7.82–4.40)	0.54
*Fixed Play Environment*	6.75 (1.90)	7.00 (3.01)	8.00 (1.90)	10.25 (1.85)	−3.25 (−6.90–0.40)	0.07
*Staff Behaviours*	14.42 (3.33)	11.42 (4.45)	14.92 (3.52)	13.02 (2.80)	−1.33 (−4.71–2.06)	0.39
*Physical Activity Training and Education*	11.33 (7.30)	8.67 (5.58)	15.33 (7.30)	16.67 (4.71)	*8.00 (−15.53–0.47)*	*0.04*
*Physical Activity Policy*	4.00 (8.94)	8.57 (10.69)	4.00 (8.94)	4.00 (8.94)	4.00 (−17.05–9.05)	0.50
Weather						
Minimum temperature (degrees Celsius, mean, SD)	10.33 (3.2)	17.83 (2.7)	8.16 (3.5)	15.32 (2.4)	1.46 (−1.05–3.97)	0.22
Maximum temperature (degrees Celsius, mean, SD)	21.72 (3.1)	29.95 (4.4)	23.53 (1.8)	31.41 (4.9)	*3.20 (0.44–5.96)*	*0.03*
UV index (mean, SD)	4.74 (0.9)	9.82 (0.9)	5.22 (0.9)	9.58 (0.6)	−0.47 (−1.33–0.40)	0.25

p-value < 0.05 is considered significant added

The maximum temperature and the EPAO subscore for educator training were found to be significantly different between groups, but there was no association when tested against the primary outcome. Precisely, the maximum temperature difference between groups was 0.03 degrees celcius (95% CI −1.40–1.33, *p* = 0.95) whereas the PA training and education was − 0.47 (95% CI −1.19–0.24, *p* = 0.37).

### Child physical activity

#### Primary outcome

Relative to children in control services, mean daily minutes of MVPA in care was significantly greater at follow-up among children attending intervention services when multiple imputation for missing data was applied (adjusted difference between groups 5.21 min, 95% CI 0.59–9.83, *p* = 0.03). These effects were also significant when complete case analysis was undertaken (adjusted difference between groups 6.11 min, 95% CI 0.54–11.68, *p* = 0.04) (Table 4). Of note, 15 children in one service spent part of 1 day of the week off site on a field excursion. However, after removing their data, the difference between groups for mean daily minutes of MVPA in care remained significant (adjusted difference 6.08 min, 95% CI 0.38–11.77), *p* = 0.04).

**Table 4:** Outcomes by group (adjusted for age, sex, and outdoor free-play duration at follow-up)

	Intervention		Control		Intervention-Control (complete case)		Multiple imputation (missing data at both time points)	
	Baseline *N* = 161	Follow-up *N* = 135	Baseline *N* = 217	Follow-up *N* = 222	Adjusted difference between groups (95% CI)	*p*-value	Adjusted difference between groups (95% CI)	*p*-value
Primary outcome								
Mean daily minutes of physical activity in care (sd.)								
MVPA (ICC-0.09)	58.53 (21.19)	58.70 (20.10)	51.72 (17.39)	52.21 (16.81)	6.11 (0.54– 11.68)	0.04	*5.21 (0.59–9.83)*	*0.03*
Secondary outcomes								
Mean daily minutes of physical activity in care (sd.)								
Vigorous PA	23.54 (11.01)	23.06 (10.34)	19.80 (8.50)	19.82 (8.38)	2.59 (−0.91–6.09)	0.13	2.09 (−0.56–4.75)	0.12
Moderate PA	34.98 (11.35)	35.64 (11.37)	31.92 (9.67)	32.40 (9.42)	3.52 (1.19–5.86)	< 0.01	*3.12 (0.91–5.33)*	*< 0.01*
Light PA	54.96 (14.17)	55.86 (13.37)	53.27 (11.55)	54.41 (11.70)	2.70 (−2.61–8.01)	0.27	2.44 (−1.56–6.45)	0.23
Total PA	113.49 (32.19)	114.56 (30.88)	104.99 (26.77)	106.63 (26.09)	8.94 (−1.43–19.31)	0.08	7.75 (−0.38–15.88)	0.06
Counts per minute in care per day (total child PA in care) (sd.)								
Counts per minute (ICC – 0.10)	196.81 (64.22)	197.06 (61.40)	176.50 (52.24)	178.67 (50.40)	16.95 (−4.63–38.52)	0.11	14.25 (−2.26–30.76)	0.09
Percentage of wear time in care per day (%) (sd.)								
% MVPA (ICC −0.12)	17.56 (5.96)	17.51 (5.34)	15.27 (4.70)	15.10 (4.33)	1.78 (0.72–2.83)	< 0.01	*1.57 (0.64–2.49)*	*< 0.001*

*ICC* intra-cluster correlation

p-value < 0.05 is considered significant added

Among children with valid data in the out of care period, children attending intervention services had higher mean daily minutes of MVPA during the out-of-care period on childcare days than children attending control services (adjusted difference between groups 7.64 min, 95% CI 3.51–18.80, *p* = 0.14); however this difference was non-significant.

#### Secondary outcomes

After imputation, adjusted differences in the percentage of wear time in MVPA in care per day for children in intervention services relative to control services was 1.57% (95% CI 0.64–2.49, *p* < 0.001). For complete case analysis, the adjusted difference between groups was 1.78% (95% CI 0.72–2.83, *p* < 0.01). Total physical activity in care per day, as assessed via counts per minute, not significant at 14.25 counts per minute (95% CI −2.26–30.76, *p* = 0.09) for imputed data analysis (an effect equivalent to 7.75 min of activity across the day). Likewise, this was not significant for the completed case analysis with the adjusted difference being 16.95 counts per minute, (95% CI −4.63–38.52, *p* = 0.11), (an effect equivalent to 8.94 min of activity across the day). (Table 4).

The median number of child injuries requiring documentation in intervention services was 33.5 (range 19–71) and in control services 35.0 (range 0–80) at baseline. At follow-up, the number of child injuries was lower at 27.5 (range 13–42) for intervention services and 28.0 (range 3–40) for control services. There was no significant difference observed in the number of injuries reported across the study period between groups (*p* = 1.0).

#### Subgroup analyses

There were no subgroup interactions for the primary trial outcome by child age, sex, or baseline MVPA levels. (Table 5).

**Table 5:** Average daily MVPA subgroup analysis (adjusted for age, sex, and outdoor free-play duration at follow-up)

Subgroup	Subgroup Level	Intervention		Control		Group x subgroup	
		Baseline *N* = 161	Follow-up *N* = 135	Baseline *N* = 218	Follow-up *N* = 222	estimate (95% CI)	*p* value
Sex	Boys	64.69 (21.03)	64.85 (19.49)	57.20 (17.85)	57.13 (17.05)	2.87 (−4.69–10.43)	0.41
	Girls^a^	50.30 (18.56)	50.27 (17.88)	46.08 (14.99)	47.11 (15.00)	–	
Baseline moderate-to-vigorous activity	More active	72.77 (14.28)	65.06 (17.13)	67.15 (10.29)	58.65 (16.32)	0.82 (−6.9–8.54)	0.81
	Less active^a^	39.05 (9.97)	47.78 (18.85)	38.77 (9.15)	44.27 (13.11)	–	
Age	3 year olds	52.94 (23.56)	55.64 (21.41)	45.39 (16.39)	48.89 (16.52)	2.53 (−9.42–14.68)	0.66
	4 year olds	60.72 (19.79)	59.17 (18.02)	53.74 (17.21)	52.72 (15.93)	1.84 (−9.43–13.11)	0.73
	5 year olds^a^	67.56 (13.79)	66.72 (25.62)	59.93 (14.10)	57.15 (19.31)	–	

^a^Denotes subgroup level used as a reference for the interaction estimate

## Discussion

This study assessed the efficacy of a simple scheduling intervention in increasing the time preschool-aged children spent in MVPA while in care. The intervention was effective in increasing daily MVPA in children attending care by approximately 5 min. Further to this, enhanced physical activity during the childcare hours did not reduce physical activity levels in periods out of care, nor result in adverse effects such as injuries. Modifying the scheduling of outdoor free-play periods in childcare services may therefore, provide an effective strategy to contribute to population level improvements at child physical activity.

The findings from this study are consistent with another trial that has modified the scheduling of outdoor free-playtime to enhance child activity. The intervention trialled by Tucker and colleagues [21] combined staff training, provision of portable play equipment and four opportunities for outdoor free-play (four 30 min blocks) and found that the intervention increased children’s MVPA by 1.28 min per hour compared to control services. In addition, a pilot study conducted in Belgian preschools [42] found that by scheduling extra recesses to reduce playground density by dividing children playing at the same time, small increases in MVPA were observed. The findings from the current study are also consistent with ecological interventions in other settings, which have aimed to modify the scheduling of free-play. For example, ecological interventions conducted in schools [43, 44] have also reported increases in child physical activity and observational studies have reported an association between periods of outdoor free-play and child activity [45, 46]. Collectively such findings provide an increasing evidence base for supporting the implementation of scheduling based interventions in childcare services.

Subgroup analysis did not support a moderator of the intervention effect for age, sex, or physical activity at baseline. These findings are in contrast to other effective physical activity interventions in this setting that have reported differences in intervention effects in subgroup analysis including sex and age. Such subgroup effects have been previously observed in trials that have targeted a range of organisational, social, and environmental determinants of child activity in care. The findings suggest that, unlike such complex interventions, simple interventions targeting environmental stimuli that align with natural physical activity patterns of children may produce more equitable intervention effects for females and children of varying ages.

The recently released 24-h physical activity guidelines recommend that children accumulate 180 min of active play of which 60 min are energetic in nature. The mean daily total physical activity among children in our sample are well below the current 180 mins recommended [47] and increased, relative to control by approximately 5 min in the intervention group. The findings suggests that while the intervention may make an important contribution to achieving the new guidelines, additional intervention is likely required. The addition of other ecological interventions, such as reducing playground density in childcare services [42] where crowding is an issue, or the introduction of portable play equipment [48–52] may provide additional enhancement to the effects of intervention. Reviews have also identified a range of other policies and practices that childcare services could undertake to enhance child physical activity [53]. However, intervention in this setting alone will not be sufficient to achieve the movement guidelines. Investment in interventions across community settings and in the home is therefore warranted.

Collectively the findings of this study, and previous research [21] support the implementation of interventions to increase the frequency of opportunities for outdoor free-play. However further research is required to identify what specific types of support services may be required to assist them to do so in the long term. In the current study, anecdotally, four of the five intervention services, continued to deliver the intervention following trial completion. By contrast, in the trial by Tucker and colleagues [21], services reported difficulties in implementing four periods of outdoor free-play as part of their curriculum and at a longer follow up, implementation had ceased. A greater understanding of the barriers to implementation of such interventions reported by representative samples of childcare services are required to better assess the potential for setting wide uptake of the intervention. During this study, educator concerns include disruption of routines for children with behavioural challenges, additional time taken for the application of sunscreen and hats, and having to adjust the childcare curriculum. Consideration of such barriers are required if large-scale dissemination and uptake of the intervention is to be achieved.

Strengths of this study include the use of a randomized trial design, and objective measurement of child physical activity over five consecutive days. The addition of reporting child injury also complements the assessment of physical activity to allay carer safety concerns that comes with outdoor risky play [54]. However, participating families in the study were from higher educated and higher incomes brackets than the general population, which could limit the representativeness of the study. Furthermore, the study used a convenience sample, a group that may be pre-disposed to implementation of the intervention. Data collectors were not blind to group allocation, and while research assistants were instructed to limit any interactions with children or staff during data collection, the presence of research assistants may have influenced typical physical activity practices or staff child interactions in both intervention and control services. The use of other data collection methods that are less intrusive, such as light sensors or global positioning systems [55, 56], may reduce the potential for any researcher reactivity in future trials. Lastly, data were collected over a change of seasons [37], which may have affected the number of days available for outdoor free-play. Future studies may look into conducting their data collection over a period of 2 years to remove the effect of season change on the availability of outdoor free-play time.

## Conclusions

Low levels of physical activity amongst preschool-aged children continue to be of concern. The study found that modest but meaningful improvements in child activity in this setting can be achieved with simple changes to scheduling of outdoor play periods. Future research identifying optimal methods to support implementation of the intervention is warranted.

## Additional file

Additional file 1: Accelerometer items to report. (DOCX 13 kb)

## Abbreviations

CI: Confidence intervals
ICC: Intra-class correlation
MVPA: Moderate-to-vigorous physical activity
PA: Physical activity
sd.: standard deviation
SES: Socio-economic status
