# Supplementary material for: Impact of scheduling multiple outdoor free-play periods in childcare on child moderate-to-vigorous physical activity: a cluster randomised trial
Source: Int J Behav Nutr Phys Act. 2018 Apr 4;15:34. doi: 10.1186/s12966-018-0665-5 (PMC5883422; doi:10.1186/s12966-018-0665-5)
Supplement: Supplementary file 1 — Accelerometer items to report. (DOCX 13 kb) [file 12966_2018_665_MOESM1_ESM.docx]

Additional file

Appendix 1: Accelerometer items to report

| Brand and model of accelerometer used | ActiGraph GT3x+ |
| --- | --- |
| Epoch length used for data collection and analysis | Data collection: 15 s epochs |
|  | Data analysis: 5s epochs |
| Placement of accelerometer and side of body | Right hip, anterior axillary line |
| Number of participants receiving accelerometer | Baseline: 379 participants |
|  | 12 weeks post-intervention: 348 participants |
| Days of data collected at each time point | Baseline: In-Care: 5 days (arrival to 3 pm)  All-day: 7 days, all waking hours |
|  | Post-intervention: In-care (arrival to 3 pm)  All-day: 7 days, all waking hours |
| Criteria for defining non-wear of accelerometer | ≥20 min of continuous 0s |
| Number of valid days and number of minutes per day of accelerometer data needed to be included in analysis | InCare: wear time for at least 50% of the school day and any day present at Childcare |
|  | All day: ≥3 days with ≥6 hours of wear-time |
| Accelerometer data PA outcome of interest and the interpretation method | Time in moderate-to-vigorous PA (MVPA) in-care, percent MVPA (adjusted for wear time) , total PA (counts per minute) |
|  | Pate 2006 cut-points, Sedentary (<25 counts/15 seconds); light (25–419 counts/15 seconds); MVPA (≥420 counts/15 seconds);  and total (≥200 counts/15 seconds) |
| Number of participants non-compliant or who had accelerometer malfunction issues | 3 participants did not meet wear-time at baseline  1 participant did not meet wear time at follow-up |

As advised in

Montoye, A. H., et al. (2016). "Reporting accelerometer methods in physical activity intervention studies: a systematic review and recommendations for authors." Br J Sports Med: bjsports-2015-095947.
